# Craniopharyngioma and the Third Ventricle: This Inescapable Topographical Relationship

**DOI:** 10.3389/fonc.2022.872689

**Published:** 2022-03-22

**Authors:** José María Pascual, Ruth Prieto

**Affiliations:** ^1^ Department of Neurosurgery, La Princesa University Hospital, Madrid, Spain; ^2^ Department of Neurosurgery, Puerta de Hierro University Hospital, Madrid, Spain

**Keywords:** craniopharyngioma, hypothalamus, papillary craniopharyngioma, pituitary infundibulum, third ventricle, third ventricle floor

## Introduction

### The Third-Ventricle Craniopharyngioma Surgical Challenge, a Hot Topic in Frontiers in Oncology

Craniopharyngiomas (CPs) are widely categorized as a group of benign epithelial tumors developed around the region of the sella turcica (1). However, from a surgical viewpoint, CPs have consistently been considered a particularly challenging intracranial tumor, owing to their close relationship to the hypothalamus and their biological infiltrating behavior (2, 3). The exceedingly heterogeneous CP topography and their usual extension into the third ventricle (3V) remain significant impediments to standardize a common management (4). Consequently, a wide array of surgical approaches, resection philosophies and adjuvant treatment guidelines have been employed and advocated, with no clear consensus being reached among authors (5, 6).

In the last decade, the experience gained from using the endonasal endoscopically assisted approach (EEA) has made this technique the gold standard for treating most sellar and suprasellar CPs (7, 8). Nevertheless, a high rate of CPs develop primarily at the infundibulo-tuberal region of the third ventricle floor (3VF) and expand within the 3V, above an intact pituitary gland and stalk (9, 10). The pervasive problem of identifying a “safe” cleavage plane through the tenacious adherence between the CP and the adjacent hypothalamus has remained the major obstacle for radical excision of infundibulo-tuberal CPs employing the EEA (11, 12) This difficulty becomes particularly delicate when dealing with papillary CPs (PCPs) having a strict or intrinsic 3V location, for which the EEA was originally regarded unsuitable and too risky, as it forced breaking through the seemingly functional 3VF, a maneuver that could potentially cause irreversible hypothalamic sequelae (13, 14). Therefore, transcranial-transventricular methods of approach have been employed to remove these intraventricular CPs, usually through the corpus callosum or by opening the lamina terminalis, a choice based on an accurate preoperative MRI diagnosis of the strict 3V topography (15–17).

*Frontiers in Oncology’s* research topic, “*Advances in craniopharyngiomas: from physiology to clinical management*” gathers a series of papers specifically focused on the clinical assessment and surgical treatment of the subgroup of intrinsic or strictly 3V CPs (18–21). The studies by Deopujary et al. and Zhao et al. direct their attention on the physiological and neuropsychological disturbances derived from the hypothalamic injury caused by resecting intra-3V CPs (18, 19). The feasibility of combining the extended endonasal endoscopically assisted approach (EEEA) with a trans-lamina terminalis (TLT) access to successfully remove strictly 3V CPs is the major objective of Cao et al. and Zhou et al. papers (20, 21). Potentially, a paradigm shift in the surgical method of choice to remove 3V CPs might occur from these studies, from the dominant use of transcranial-transventricular routes to a generalized use of the EEEA plus TLT (5, 22). Beyond that, however, all these works can shed light on the specific pathological features and hypothalamic alterations associated with infundibulo-tuberal and strictly 3V CPs, two topographical categories which need to be differentiated from the rest of sellar/suprasellar lesions (10).

### The Craniopharyngioma-3V Relationship: Anatomical and Neuroradiological Evidence

For the last decades, our team has analyzed the anatomical relationships between CPs and the adjacent 3V that can be accurately defined in well-characterized individual CP reports (12, 23–25). Thus far, our research involves the exam of more than 1,000 autopsied CP specimens from non-operated patients and the correlation between the CP-3VF relationship observed on preoperative MRI scans and surgical findings in about 2,700 CP patients. This body of evidence has enabled us to differentiate four basic CP-3V relationships, which depend on the original site of CP development (beneath, within or above the 3VF) and the 3VF distortion pattern (3VF displaced upwards, expanded by the tumor or invaded by the tumor) (23, 24). In contrast to other CP classification methods, our scheme focuses on the way the 3V is affected by the lesion. Acknowledging the type of CP-3V relationship allows to define a set of clinical-pathological CP features specific to each topographical category. Even more importantly, a 3V-centered scheme also helps to predict the extension and strength of the CP-hypothalamic attachment, which largely determines the risk of radical removal (12, 26, 27). The works by Deopujarny et al., and Cao et al., whose surgical series encompass more than 800 CPs, have verified to a large extent our topographical concepts (18, 20). The attention that should be given to the hypothalamic symptoms caused by 3V CPs and the types of CP-hypothalamus attachment associated with this topography is the main message we wish to emphasize from these studies published in *Frontiers in Oncology*.

### Craniopharyngiomas With a Primary Intra-3V Development: Types and Distinctive Features

**Figure 1:**
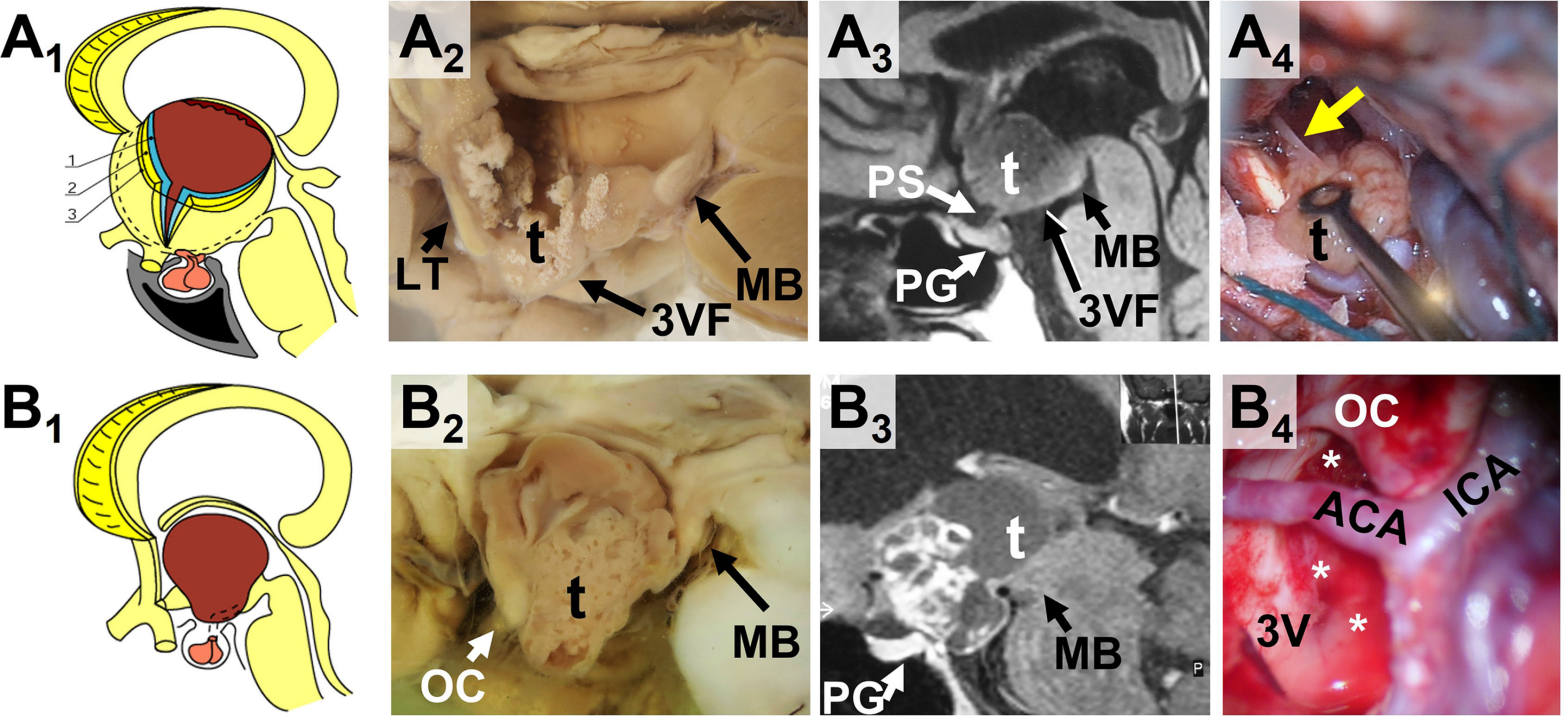
Craniopharyngiomas with a primary third ventricle development (3V CPs): comparison between the two major 3V CP topographies. **(A_1_-A_4_)** The strictly 3V topography. The upper row panels show the anatomical **(A_1_)**, gross pathological **(A_2_)**, neuroradiological **(A_3_)** and surgical **(A_4_)** evidence for the strictly or truly 3V CP topography. **(A_1_)** Anatomical sketch showing a CP wholly confined within the 3V cavity, above an intact third ventricle floor (3VF). The three layers that forms the 3VF, ependyma (1), the 3VF neural tissue including the median eminence and infundibulum (2) and the pia mater (3) remain intact below the tumor, originated at a subependymal position. **(A_2_)** Midsagittal section of a gross pathological CP specimen with a strict 3V topography. A tenuous, but still present 3VF layer covers the basal boundary of the tumor. LT: Lamina terminalis; MB: mammillary body (Original brain specimen from the Vienna anatomical-pathological collection, housed at the Narrenturm). **A_3_** T1-weighted MRI midsagittal scan of a strictly 3V CP diagnosed in a 46-year-old female patient showing headache, blurred vision with homonymous left inferior quadrantanopia, diabetes insipidus and a depressive disorder for 1 year. Notice how this largely solid tumor of the papillary type (t) is entirely confined within the 3V, above an intact 3VF and pituitary stalk (PS) and gland (PG). **(A_4_)** Intraoperative image showing the narrow, pedicle-like CP attachment (yellow arrow) to the 3VF of a strict 3V papillary CP. **(B_1_-B_4_)** The infundibulo-tuberal or not strictly 3V topography. The lower row panels show the anatomical **(B_1_)**, gross pathological **(B_2_)**, neuroradiological **(B_3_)** and surgical **(B_4_)** evidence for the not strictly 3V CP topography, also known as infundibulo-tuberal. **(B_1_)** Anatomical sketch showing a CP replacing the 3VF and largely occupying the 3V cavity. The lesion has primary developed at the neural layer of the 3VF (infundibulum and/or tuber cinereum) replacing progressively the 3VF while expanding into the 3V cavity. **(B_2_)** Midsagittal section of a gross pathological CP specimen with an infundibulo-tuberal topography. The lower pole of this chiefly solid 3V CP (t) protrudes towards the suprasellar cistern after replacing the region of the infundibulum-tuber cinereum. The mammillary bodies (MB) are the only remaining structures of the 3VF. OC: optic chiasm (Original brain specimen from the Vienna anatomical-pathological collection, housed at the Narrenturm). **(B_3_)** T1-weighted MRI midsagittal scan of an infundibulo-tuberal CP diagnosed in a 32-year-old male patient showing blurred vision with bitemporal hemianopia, progressive obesity, hyperphagia, unmotivated rage episodes, and memory disturbances for the last months. Notice how this large, solid-cystic CP of the adamantinomatous type (t) has replaced the 3VF and the infundibulum-pituitary stalk, occupying both the 3V cavity and the suprasellar cistern, above an intact pituitary gland (PG). **B_4_** Intraoperative image of the 3V after total removal of the tumor through a trans-lamina terminalis approach. Notice the hemorrhagic border of the breached 3VF (white asterisks) corresponding to the ring-like band of tight attachment between the hypothalamus and the central CP region, often found for infundibulo-tuberal or not strictly 3V CPs. ACA, Anterior communicating artery; ICA, Internal carotid artery. OC, optic chiasm.

Two major CP topographies primarily originate within the neural tissue of the infundibulum and tuber cinereum, the components of the 3VF: the not strictly intraventricular or infundibulo-tuberal, which expands within the 3VF itself and replaces it progressively; and the strictly intraventricular, which, owing to its subependymal origin, mostly expands within the 3V cavity above a stretched but anatomically intact infundibulum (9, 28) (**Figure 1**). Both types represent lesions primarily affecting the hypothalamus, which means that the tumor is partly or wholly embedded within the 3VF, often encircled by a band of non-functional gliotic tissue (10). Infundibulo-tuberal CPs constitute approximately 40% of lesions in the adult CP population. The majority belong to the adamantinomatous type (ACPs) and show the strongest and riskiest attachments to the hypothalamus (28). The scarcer subgroup of strictly 3V CPs only comprises about 5% of cases, also involves predominantly adults (92%) and largely includes lesions of the papillary type (82%). Strictly 3V PCPs characteristically present weaker, lower-risk attachments to the 3VF than not strictly intraventricular ACPs (12, 23). Interestingly, despite their more benign attachment, these PCPs with an intrinsic or strict intra-3V development very often cause a wide range of psychiatric disturbances (in up to 60% of patients), owing to the severe tumoral compression upon the hypothalamus (10, 29). These emotional, behavioral and cognitive alterations, poorly addressed in most surgical CP series, represent a true organic model of psychiatric disease of great potential relevance for elucidating the neurobiological basis of psychiatric disorders (29).

More recently, we were able to compile and analyze comprehensively the cohort of CPs with a verified strictly 3V topography (n=245), as well as the historical cohort of well-described papillary CPs published in the medical literature (n= 350) (30, 31). Although the strictly 3V topography has remained controversial throughout history, some authors considering it an exceptional, ectopic location (32), while others even argue over its validity (33), the surgical series by Depoujarny (18), Cao (20), and Zhou (21) contribute to verify this particularly challenging location, confirming the anatomical integrity of the 3VF found in numerous strictly 3V CPs in prior studies (see **Table 1**) (34–49). The optimal surgical view of the brain undersurface obtained through the EEA unequivocally show the ballooned and stretched infundibulum wrapping around the lower pole of these lesions, which stay hidden within the 3V chamber (13, 14, 40, 46). In 5 out of 6 strict CP cases in the series by Cao (83%) and 6 out of 9 in Zhou’s paper (66%), the lesions corresponded to the papillary type, percentages that fit well with the 82% rate of papillary lesions found in our systematic review (20, 21, 30). Depoujarny observed preoperatively symptoms related to hypothalamus dysfunction in 60% of their 3V CP patients overall, the most prominent being memory loss (25%), increased sleepiness (20%) and abnormal uninhibited behaviors, including hyperphagia (36%) (18). These figures also match with the rate of mental alterations in strictly 3V CPs (59%) and papillary CPs (50%) identified in our reviews (30, 31). Visual and endocrine symptoms, typical of ACPs with a suprasellar location below the 3V, were, however, rather low, in the range between 40-55% in both Depoujarny and Zhou studies (18, 21).

**Table 1:** Epidemiological, clinico-pathological and surgical characterization of third ventricle craniopharyngiomas (3V CPs) included in modern CP surgical series.

CP series/Year [ref]	No. 3V CPs/ Adults Rate	Rate 3V CPs/Total No. CPs	Histology Types	Hypothalamic/Psychic symptoms	Main Approach/GTR rate	Mortality/Postop H.I. ^†^	Recurrence/Follow-up
Yasargil et al. (34)	7	4%	NA	NA	TC: 100%	NA	NA
					100%		
	100% A	162					
Davies et al. (35)	6	NA	3 pCP	16.5%	TLT: 100%	0%	50%
	100% A		3 aCP	Psychic: 16.5%	66%	80%	8 y
Maira et al. (36)	8	11%	2 pCP	25%	TLT: 100%	12.5% (1y)	14%
	100% A	72	6 aCP	Psychic: 25%	87.5%	25%	5 y
Behari et al. (37)	6	8%	NA	33%	TC: 50%	16.6%	0%
	66% A	75		Psychic: 33%	50%	16.6%	3 y
Pascual et al. (23)	105	NA	29 pCP	55%	FTV/TC: 68%	29%	NA
	85% A		29 aCP	Psychic: 40%	55%	18%	
Sohma et al. (38)	5	NA	3 pCP	40%	TLT: 100%	0%	20%
	4 A		2 aCP	Psychic: 40%	100%	NA	5 y
Shi et al. (39)	23	8%	NA	NA	TLT: 56%	NA	NA
	NA	284			74%		
Pan et al. (15)	17	8,7%	6 pCP	47%	TLT: 100%	12%	17.5%
	15 A	195	11 aCP	Psychic: 47%	76.5%	12%	4 y
Jung et al. (16)	4	NA	4 aCP	0%	TC: 100%	0%	50%
	100% A				100%	0%	4y
Cavallo et al. (40)	12	29%	NA	16.6%	EEA: 100%	8.3%	9%
	92% A	41		NA	66.7%	18%	1y
Yu et al. (41)	24	3%	10 pCP	33.3%	TC: 62.5%	12.5%	25%
	100% > 15y	830	14 aCP	Psychic: 33.3%	79%	8%	4 y
Morisako et al. (42)	12	16.5%	2 pCP	33%	TLT: 100%	0%	0%
	Mean age: 45	72	10 aCP	Psychic: 25%	75%	25%	4 y
Zoli et al. (43)	10	NA	5 pCP	100%	EEA: 100%	0%	10%
	100% A		5 aCP	Psychic: NA	80%	20%	1 y
Nishioka et al. (44)	3	NA	2 pCP	0%	EEA: 100%	0%	0%
	2 A		1aCP	Psychic: NA	100%	33%	1 y
Mortini (45)	6	NA	NA	66.6%	TLT: 100%	0%	0%
	100% A			Psychic: 50%	100%	33%	2.5 y
Forbes et al. (46)	10	12.5%	3 pCP	40%	EEA: 100%	0%	20%
	100% A	80	7 aCP	Psychic: 20%	90%	30%	4 y
Seo et al. (47)	26	34%	11 pCP	23%	EEA: 100%	0%	3.8%
	76% A	76	15 aCP	Psychic: 19%	88.5%	NA	3y
Fan et al. (48)	26	11.5%	5 pCP	34.5%	EEA: 100%	0%	4%
	92% A	223	19 aCP	Psychic: NA	92%	34.5%	1 y
Hung et al. (49)	5	NA	5 pCP	NA	FTV: 4; TLT: 1	NA	NA
	100% A				NA		
Deopujari et al. (18)	25	4.3%	NA	60%	FTV: 56%	8%	20%
	NA	585		Psychic: 44%	40%	30%	3y
Zhao et al. (19)	17	10%	NA	NA	NA	NA	NA
	NA	173		Psychic: 47%			
Cao et al. (20)	8	5.3%	6 pCP	37.5%	EEA-TLT: 100%	0%	12.5%
	100% A	149 SS *	2 aCP	Psychic: 12.5%	100%	12.5%	1 y
Zhou et al. (21)	9	NA	6 pCP	33%	EEA-TLT	0%	0%
	100% A		3 aCP	NA	89%	0%	2.5y
Prieto et al. (30)	245	5.6%	182 pCP	65%	TLT: 41%; FTV/TC:41%	3.3% **	14.5%
	93% A	3,821	33 aCP	Psychic: 59%	52%	23%	3 y

A, adults; aCP, adamantinomatous type; CP, craniopharyngioma; EEA, endonasal endoscopic approach; FTV, frontal transventricular approach; GTR, gross total removal; H.I., hypothalamic injury^†^; NA, not available; No., number; pCP, papillary type; postop, postoperative; TC, transcallosal; TLT, trans lamina terminalis; y, years; 3V, third ventricle. *Suprasellar tumors; **Mortality rate for the tumors operated on in the most recent period between 2006-2021 (n=61).

^†^Postoperative hypothalamic injury rates include any of the following worsening of and/or sequelae: severe obesity (> 30% of BMI) with hyperphagia, severe hydroelectrolytic or autonomic disturbances, hyperthermia/poikilothermic dysfunction, gait ataxia, sphincters incontinence, psychiatric disturbances, Korsakoff-like memory defects and/or cognitive decline, all preventing autonomous life.

### The Combined EEA-Translamina Terminalis Approach for Strictly 3V CPs: A Promising Surgical Strategy

The controversy about what should be the optimal surgical strategy for strictly 3V CPs has remained unresolved ever since. The complex problem of dealing with the CP-hypothalamus plane of adherence within the 3V under a good direct view has stimulated the use of multiple transcranial routes, mainly the frontal-transventricular, the transcallosal and the translaminar-terminalis (3, 5, 34, 36). Notably, Depoujarny found strong adherences between the CP capsule and the 3VF/3V walls in 36% of cases, mainly among pure cystic lesions in which the tumor capsule had merged with the 3V boundaries (18). These high-risk adherences in strictly or largely 3V CPs more often develop in the adamantinomatous type (58%) than in the papillary one (25%), the latter usually presenting either a small pedicle-like attachment or a sessile, flat patch adherence to the infundibulum (23, 31). Strong CP-hypothalamic attachments are the main obstacle precluding a safe radical removal of the lesion, a goal only reached in 40% of Depujany’s series employing transcranial procedures (18). The more accurate assessment of the CP-3VF relationship achieved through the EEA over these open craniotomy-transventricular procedures, has changed the surgical paradigm towards the standard use of this approach to safely remove CPs involving the 3V (7, 8, 13, 40). Now, the expertise gained with the use of the EEEA allowed pituitary surgeons to incorporate the translaminar terminalis corridor to the technique to successfully remove strictly 3V CPs without mortality, as shown in the series by Cao (87.5% gross total removal, GTR) and Zhou (89% GTR) (20, 21). Accordingly, should this combination of EEEA plus TLT technique be considered the definitive method capable of overcoming the impediment of CP adherence and/or infiltration into the hypothalamus intrinsic to intra-3V development? (26, 28, 33, 50).

In our 2004 comparative analysis of the surgical approaches employed to remove intraventricular CPs up to that date, all performed through open craniotomies, we found that the TLT approach was superior to the others (transcallosal and frontal-transventricular) in terms of null postoperative mortality (23). Notably, partial degrees of tumor removal yielded poorer postoperative outcomes than total excisions, an apparently paradoxical result highlighting the damaging effect that unsuccessful attempts to dissect tight CP-hypothalamic adhesions had on the ultimate clinical outcome. The results of this research may be cautiously extrapolated to define the current indications for total removal of strictly 3V CPs employing the trans-infundibular and translamina-terminalis corridors through the EEEA. Undoubtedly, in expert hands this procedure offers the great advantage over transcranial methods of allowing an easier sharp dissection of the CP-hypothalamic plane of adherence from the initial stages of surgery (13, 20, 40). It also ensures the preservation of the hypothalamus and chiasm blood supply through basal perforating vessels, which usually remain hidden from view when employing transcranial approaches. Avoiding mechanical and ischemic injuries to the hypothalamus caused by forceful blind pulling maneuvers on the intra-3V tumor bulk is essential for the postoperative improvement of psychiatric and neuropsychological disturbances, as is shown in the study by Zhao (19, 51). Nevertheless, the type of CP-hypothalamic attachment is the crucial factor determining the possibility of eventually accomplishing a successful total removal (4, 26).

## Concluding Remarks

The infiltrative nature of CPs developed at the infundibulo-tuberal region, with finger-like tumor extensions protruding into the adjacent hypothalamus has been repeatedly confirmed on histological studies of CP boundaries (15, 26, 28, 33, 52). As is rightly noted by Depujarny, poorer clinical outcomes have been reported for CP patients showing a breached 3VF after radical removal of 3V CPs tightly attached to the 3VF (18, 36, 53, 54). Psychiatric disturbances due to hypothalamic injury can be truly devastating for the personal autonomy and social integration of CP patients (10, 55). Consequently, not all strict 3V CPs should undergo radical removal (6, 10). Regarding this, it is worth mentioning the lack of reliable information about the actual prevalence of long-term neuropsychiatric disturbances in large surgical series employing the EEA. The neuropsychiatry inventory-questionary (NPI-Q) used in the study by Zhao, taking into account the six fundamental categories of psychological disorders related to hypothalamic injury by CPs (emotional control loss, abnormal moods, odd behavioral changes, memory defects, dementia-like cognitive impairment; and/or psychotic symptoms), could well be incorporated into the standard battery of clinical tests to assess the postoperative long-term outcome of CP patients (19, 29). The concept of “maximum safe resection”, which prioritizes the preservation of hypothalamic functions and psychological autonomy over the completeness of resection should guide surgical actions when dealing with such a complex lesion as a 3V CP, regardless of how sophisticated or technologically well-equipped the surgical procedure might be (10, 18).

## Author Contributions

Conception and design: JP. Acquisition of data: JP, RP. Analysis of data: JP, RP. Drafting the article: JP. Critically revising the article: RP. All authors contributed to the article and approved the submitted version.

## Conflict of Interest

The authors declare that the research was conducted in the absence of any commercial or financial relationships that could be construed as a potential conflict of interest.

## Publisher’s Note

All claims expressed in this article are solely those of the authors and do not necessarily represent those of their affiliated organizations, or those of the publisher, the editors and the reviewers. Any product that may be evaluated in this article, or claim that may be made by its manufacturer, is not guaranteed or endorsed by the publisher.

## Abbreviations

ACP, adamantinomatous craniopharyngioma; CP, craniopharyngioma; MRI, Magnetic Resonance Imaging; PCP, Squamous-papillary craniopharyngioma; 3VF, third ventricle floor; 3V, Third ventricle.
